# Ready-to-use binder-free Co(OH)_2_ plates@porous rGO layers/Ni foam electrode for high-performance supercapacitors

**DOI:** 10.1039/d1ra08683a

**Published:** 2022-03-24

**Authors:** Mustafa Aghazadeh, Hamzeh Forati Rad, Ramin Cheraghali

**Affiliations:** Nuclear Fuel Research School, Nuclear Science and Technology Research Institute (NSTRI) Tehran Iran Mustafa.aghazadeh@gmail.com; Department of Chemistry, Islamic Azad University Saveh Branch Saveh Iran

## Abstract

In this work, an outstanding nano-structured composite electrode is fabricated through the co-deposition of Co(OH)_2_ nanoplates and porous reduced GO (p-rGO) nanosheets onto Ni foam (NF). Through field emission scanning electron microscopy and transmission electron microscopy observations, it was confirmed that porous reduced graphene oxide sheets are completely wrapped by uniform hexagonal Co(OH)_2_ plates. Due to the unique architecture of both components of the prepared composite, a high surface area of 234.7 m^2^ g^−1^ and mean pore size of 3.65 nm were observed for the Co(OH)_2_@p-rGO composite. The constructed Co(OH)_2_@p-rGO/NF composite electrode shows higher energy storage capability compared to that of Co(OH)_2_/NF and p-rGO/NF electrodes. The Co(OH)_2_/NF electrode shows specific capacitances of 902 and 311 F g^–1^ at 5 and 30 A g^–1^, while the Co(OH)_2_@p-rGO/NF electrode delivers 1688 and 1355 F g^–1^ under the same current loads, respectively. Furthermore, when the current load was increased from 1 to 30 A g^–1^, 74.5% capacitance retention was observed for the Co(OH)_2_@p-rGO/NF electrode, indicating its outstanding high-power capability, while the Co(OH)_2_/NF electrode retained only 38.5% of its initial capacitance. The fabricated Co(OH)_2_@p-rGO/NF//rGO/NF ASC device shows an areal capacitance of 3.29 F cm^−2^, cycling retention of 91.2% after 4500 cycles at 5 A g^−1^ and energy density of 68.7 W h kg^−1^ at a power density of 895 W kg^−1^. The results of electrochemical tests prove that Co(OH)_2_@p-rGO/NF exhibits good performance as a positive electrode for use in an asymmetric supercapacitor device. The prepared porous composite electrode is thus a promising candidate for use in supercapacitor applications.

## Abstract 2

Fabrication mechanism of a ready-to-use Co(OH)2@p-rGO/NF electrode: (a) base generation step, (b) electrophoretic deposition of rGO onto NF and (c) chemical formation of Co(OH)2 on rGO.

## Graphical abstract

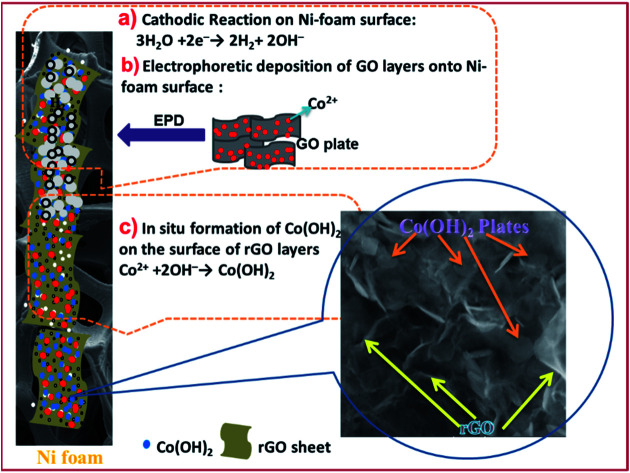

## 1. Introduction

Advanced materials with outstanding electrochemical characteristics are undoubtedly desirable to guarantee the current growth of highly effective energy storage devices.^1^ In particular, supercapacitors (SCs), also known as electrochemical capacitors, have gained more attention than batteries due to their high storage capability (*i.e.*, fast discharge time; SC: 1–10 s *vs.* lithium ion battery: 10–60 min) and excellent cyclability (SC > 30 000 h *vs.* battery > 500 h).^2–4^ There are three categories of supercapacitors based on their charge storage mechanism, which are: (i) electric double-layer capacitors (EDLCs), (ii) pseudocapacitors (PCs) and (iii) hybrid capacitors. EDLCs store charge by physical charge separation at the electrolyte/electrode interfacial area rather than *via* faradaic reactions. PCs can restore charge not only *via* an EDLC mechanism but also *via* fast electron transfer reactions that result in more charge being stored by PCs in comparison to EDLCs. Hybrid capacitors contain both EDLC and PC materials in one electrode material.^5^ Up to now, various electrode materials have been used for supercapacitor applications, in three categories: carbon materials with a high surface area (like carbon nanotubes, mesoporous carbon, graphene oxide, activated carbon, graphene, *etc.*), conductive polymers (such as polythiophene, polypyrrole, *etc.*) and transition metal-based nano-materials (like NiO, MnO_2_, Ni_3_S_2_, Co_3_O_4_, Ni(OH)_2_, Co(OH)_2_, *etc.*).^6,7^ Many porous surface modified materials have also been developed that exhibit excellent charge storage performance for supercapacitors.^8,9^ The enhancement in the specific capacitance and energy density of porous structures has been mainly attributed to an improvement in their wettability, which results in a higher useable surface area and lower internal resistance.^10,11^ Furthermore, the effects from surface modification become more marked at higher discharge rates, at which the internal resistance has a greater impact on the energy delivery.^8,12–14^ In this regard, the most important impact results from the mesoporous features of the modified porous materials.^14–16^ The macropores (or large mesopores) serve as a solution buffering reservoir to minimize the diffusion distance to the connected mesopores, facilitating mass transport, and also reducing the volume change during charge/discharge cycling, ensuring high rate capability and cycling performance.^15–17^ Nanostructured materials have been used as novel electrode support materials in catalysis, next-generation electronics, photonics, energy storage and conversion, owing to their stable porous architectures, large heterointerfaces and exceptional specific surface area.^18–24^

Among different electrode materials, cobalt hydroxide (Co(OH)_2_) has a large theoretical capacity of around 3460 F g^–1^ within a potential window of 0.6 V, however, its SC application is restricted by low electrical conductivity and large structural deterioration over successive GCD cycles.^25–27^ In this regard, reduced graphene oxide (rGO) has been demonstrated to be an ideal conductive candidate due to its high surface area as well as high conductivity in electrochemical applications.^28^ For example, a CoAl-layered double hydroxide nanosheets/RGO composite has been manufactured *via* the deposition of nitrate-intercalated CoAl-LDH nanosheets on the surface of rGO nanosheets *via* electrostatic attraction between the negative charge of graphene oxide and positive metal cations.^29^ The fabricated composite electrode exhibited 1296 F g^–1^ at a current load of 1 A g^–1^ and 90.5% capacity retention after 1000 cycles at a high discharge load of 15 A g^–1^. Lim *et al.*^30^ synthesized a hollow rGO@NiCo hydroxide composite *via* a hydrothermal route and the prepared electrode material showed a capacitance of as high as 518.4 F g^–1^ at 0.5 A g^–1^ and 97.6% stability after 3000 cycles at 2 A g^–1^. Jeong *et al.* reported the one-pot synthesis of a thin Co(OH)_2_ nanosheets on graphene material and its high activity as a capacitor electrode, where the fabricated composite electrode exhibited a specific capacitance of 960 F g^−1^ at a current density of 10 A g^−1^. In addition, after 5000 charge/discharge cycles, the Co(OH)_2_/graphene composite retained 93.4% of its initial specific capacitance at a current density of 30 A g^−1^.^31^

A NiCo-LDH/rGO composite was synthesized *via* a simple one-stage solvothermal route, wherein the fabricated electrode delivered a specific capacitance of 1911.1 F g^–1^ at a current load of 2 A g^–1^, and 74% cyclability over 1000 charge/discharge cycles at a current load of 20 A g^–1^.^32^ A hydrotalcite-like α-Co(OH)_2_/graphene suspension was also fabricated by Cheng *et al.*, with the hybrid material exhibiting a high specific capacitance of 567.1 F g^−1^ at 1 A g^−1^, while a better rate capability and stability were achieved compared to those of pristine and single exfoliated α-Co(OH)_2_.^33^

Co(OH)_2_ nanoparticles have been also anchored on the surface of rGO nanoflakes *via* a hydrothermal route, with the prepared material exhibiting a specific capacitance of 235.20 F g^–1^ at 0.1 A g^–1^ and high stability (*i.e.* approximately 90% capacity retention after 2000 cycles at 1 A g^–1^).^34^ In most of these reports, two-step processes were used to fabricate the hydroxide/rGO composite electrode.^35–38^ In fact, in most reports, the carbon component of the composite was firstly deposited as a thin film on the current collector *via* electrophoretic deposition, and the metal oxide (hydroxide) component was then coated/synthesized on the carbon/current collector *via* chemical or electrochemical procedures.^36,37^ Also, in some studies, it has been reported that the metal hydroxide component is firstly prepared on the substrate and then the carbonaceous part is electrophoretically deposited as a top layer.^39–41^ The electrophoretic deposition (EPD) route is a feasible and cost-effective technology that has been successfully used for GO precipitation due to its simple deposition procedure, thickness controllability, and simplicity of large scale production.^42,43^ Electrochemical cathodic deposition (ECD) has also been used as a facile route for the preparation of metal hydroxide nanostructures.^44–46^ For example, it has been reported that electrochemical deposition is a facile procedure for the preparation of nanocomposites such as Co(OH)_2_/CNTs, RGO/CoNi LDH and NiS/N-doped graphene.^47,48^ Herein, we introduce a one-pot and simultaneous EPD/ECD procedure for the fabrication of a cobalt hydroxide/carbonaceous composite embedded on Ni foam for the first time. In this platform, both components of the composite are simultaneously deposited onto the current collector, resulting in better physical contact between the components, enhanced interface area and better conductivity. The synthesized composite delivers a high supercapacitive performance. Using this strategy, a simple co-embedding procedure is reported for the synthesis of cobalt hydroxide@porous reduced graphene oxide on Ni foam (*i.e.* Co(OH)_2_@p-rGO/NF electrode) from GO dispersed in an aqueous solution of cobalt nitrate. For comparison, pristine Co(OH)_2_/NF and p-rGO/NF electrodes were also fabricated *via* ECD and EPD routes, respectively. To the best of our knowledge, there is no report on the one-step electrodeposition of a Co(OH)_2_@p-rGO nanocomposite. The fabricated materials were characterized *via* X-ray diffractometry (XRD), field emission electron microscopy (FE-SEM), Brunauer–Emmett–Teller (BET), transition electron microscopy (TEM), energy-dispersive X-ray spectroscopy (EDS), Raman spectroscopy and Fourier-transform infrared (FT-IR) spectroscopy analyses. The charge storage capabilities of fabricated pristine Co(OH)_2_/NF and Co(OH)_2_@p-rGO/NF electrodes were analyzed by electrochemical evaluation of cyclic voltammetry (CV), electrochemical impedance spectroscopy (EIS) and galvanostatic charge–discharge (GCD) measurements and their performances compared.

## 2. Materials and methods

### 2.1. Materials

Graphite powder (<20 μm, Qingdao Graphite Co. Ltd., China), KOH (99%, Sigma-Aldrich), H_2_SO_4_ (98%, Sigma-Aldrich), HNO_3_ (65%, Sigma-Aldrich) and cobalt nitrate hexahydrate (Co(NO_3_)_2_ 6H_2_O, Sigma-Aldrich) were attained. K_2_Cr_2_O_7_ (99.3%), and H_2_O_2_ (30%) were purchased from Merck Company. All the chemicals were utilized as-received. Deionized (DI) water with a resistance of 16.1 MΩ was utilized for the preparation of solutions.

### 2.2. Synthesis of porous graphene oxide (GO)

Typically, GO was prepared based on a previously reported route.^30,31^ In a 500 mL flask containing an acidic solution of 200 g of H_2_SO_4_, 50 g of HNO_3_ and 30 g of DI water, 15 g of KMnO_4_ was added to the flask under sonication. Then, 4 g of graphite powder was blended to the as-prepared solution and refluxed for about 3 h at 100 °C and 150 °C, correspondingly (heating rate of 2 °C min^−1^). After the attained mixture cooled to room temperature, 20 mL of hydrogen peroxide (30%) was gently added in such a way that a uniform black suspension was attained, which was then washed with DI water to attain neutral pH (∼7). Then, the resulting black homogenous suspension was gathered by centrifugal precipitation and dehydrated at 70 °C overnight. Finally, the suspension was exfoliated by exposure to ultrasound waves for 2 h, which by this strategy the bulk GO powder was transformed into exfoliated GO sheets.

### 2.3. Fabrication of electrodes

**Fig. 1:**
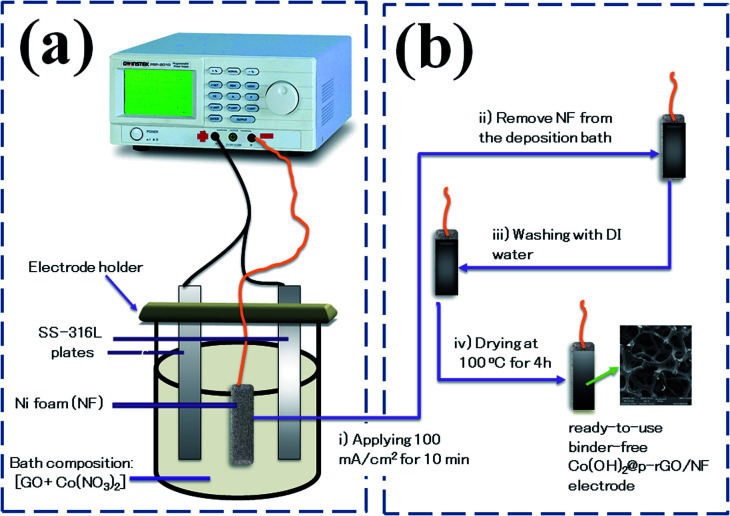
Schematic of fabrication Co(OH)_2_@rGO onto Ni-Foam including (a) electrochemical set-up and (b) deposition procedure.

Typically, a two-electrode electrochemical set-up was used in the electrodeposition runs. The negative electrode was a Ni foam/or Ni foil (surface area = 2 cm^2^) located between two equivalent steel plates as positive electrodes (surface area = 4 cm^2^). The electrodeposition set up is shown in Fig. 1a. The electrodeposition bath was prepared as follows: first, 40 mg of the synthesized porous graphene oxide was directly dispersed by sonicating the probe in 100 mL of DI water for 30 min. To get better GO dispersion, the sonication treatment was performed at 70 °C. Then, 291 mg of cobalt nitrate (10 mM of Co(NO_3_)_2_·6H_2_O) was added to the graphene oxide suspension and the mixture was agitated for 12 h. The resultant mixture was used as the deposition media for the precipitation of composite material onto Ni foam or Ni sheet. To prepare the cobalt hydroxide/p-rGO hybrid material, a current load of 100 mA cm^−2^ was used in electrolyte media containing both nickel ions and graphene oxide precursors (Fig. 1b). The duration of the electrodeposition and the temperature of the electrolyte were adjusted to 10 min and 60 °C, respectively. After electrodeposition, the Ni foam cathode was rinsed several times with DI water and dehydrated at 100 °C for 4 h. This cathode was labelled as the Co(OH)_2_@p-rGO/NF electrode and used as a binder-free working electrode in all electrochemical tests. To prepare a composite powder, Ni sheet was used instead of nickel foam, and the same electrodeposition run was done. After deposition, the Ni sheet was removed from the electrolyte media and washed numerous times with DI water. The precipitated film was then separated from the Ni cathode, and the wet deposit was calcined in the oven at 100 °C for 4 h. The final dehydrated powder was labelled as Co(OH)_2_@p-rGO and fully analyzed using different techniques. Furthermore, the electrodeposition runs were performed in p-GO-free deposition media onto both Ni foam and Ni sheet cathodes. A similar washing and drying process was applied on these both the cathodes and the final products were labelled as the pristine Co(OH)_2_/NF electrode and pristine Co(OH)_2_ powder.

### 2.4. Characterization instruments

N_2_ adsorption–desorption isotherms at 77 K were used to estimate the specific surface area of the electrodes using a Micrometrics ASAP 2010 system. Raman spectra were recorded in the range of 200 cm^−1^ to 2000 cm^−1^ using a RenishawinVia Raman microscope equipped with an Ar^+^ laser (*λ* = 514.5 nm). The size, shape and microstructure of the fabricated samples were detected by field-emission scanning electron microscopy (FE-SEM, Mira 3-XMU with an applied potential of 40 kV) and transmission electron microscopy (TEM, Zeiss EM900 with an accelerating potential of 80 kV). The EDS analyses of samples were carried out by Mira 3-XMU FE-SEM at 100 kV. The crystal structures of the fabricated samples were detected *via* X-ray diffractometry (XRD, X'Pert PRO diffractometer) using CuK_α_ radiation. FT-IR spectra of the samples were recorded using a Nexus 670 FT-IR spectrometer in the wavenumber range of 400 to 4000 cm^−1^.

### 2.5. Electrochemical measurements

#### 2.5.1. Electrochemical study in a three-electrode system

CV, GCD and EIS profiles were studied using a CHI 666D electrochemical system. A three-electrode configuration was used for the CV, EIS and GCD tests, where the fabricated electrodes, Ag/AgCl and platinum foil were used as the working, reference and auxiliary electrodes, respectively. All the electrochemical tests were carried out in an aqueous solution of 2 M KOH. The mass loads of Co(OH)_2_ and Co(OH)_2_@p-rGO onto NF were 5.9 mg and 4.2 mg, respectively. CVs of the prepared electrodes were recorded in 2 M KOH electrolyte within the potential window of −0.2 and 0.6 V *vs.* Ag/AgCl at scan rates of 2, 5, 10, 20, 50, 75, 100, 200 and 300 mV s^−1^. The capacitance values (F g^−1^) of both electrodes were calculated from the CVs using the following formula:^49^1
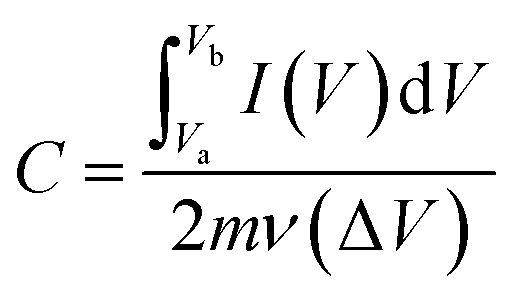
where *C* (F g^−1^) is the capacitance value, *I* (V) is the current delivered by the electrode within the potential scan, Δ*V* is the investigated potential difference between the highest and lowest potential, *m* is the weight of active materials in g, *ν* is the potential sweep rate in V s^−1^ and *I* (V) is a current signal within the voltage sweep. GCD tests were also recorded at current loads of 1, 3, 5, 10, 15, 20 and 30 A g^−1^ in the potential window of −0.1 to 0.5 V *vs.* Ag/AgCl. The following equation was utilized to estimate the specific capacitances of the fabricated electrodes from GCD data:^50^2
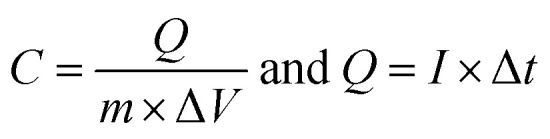
where *C* (F g^−1^) is the measured capacitance of the working electrode, *I* is the current load applied in the GCD tests (A), Δ*V* is the potential window (0.6 V), Δ*t* is the time of the discharge cycle (s) and *m* is the weight of the deposited material (g). Electrochemical impedance spectroscopy (EIS) investigations were conducted in the frequency range of 100 kHz to 0.01 Hz with an amplitude signal of 5 mV.

#### 2.5.2. Fabrication and electrochemical study in a two-electrode system

A simple asymmetric SC was fabricated using Co(OH)_2_@p-rGO and reduced GO as the positive and negative electrode, respectively. The details of the fabrication progress are as follows: the galvanostatic discharge experiments of Co(OH)_2_@p-rGO and rGO were carried out at the same current density in a three-electrode system to estimate their specific capacitances. The mass ratios of the active materials was obtained using the following relationship:^52,53^

3 *m*^+^/*m*^–^ = (*C*^–^ ⨯ Δ*V*^–^)/(*C*^+^ ⨯ Δ*V*^+^)

where *C* is the specific capacitance observed from the GCD measurements. In this work, the mass loadings of the positive (*i.e.* Co(OH)_2_@p-rGO) and negative (rGO) materials were around 6.5 mg and 4 mg, respectively. The specific capacitance of a single electrode in the ASC was calculated using the following relationship:^52,54^

4 *C*_s_ = 4 × *I* × Δ*t*/(Δ*V* × *m*′)

wherein *m*′ (g) is the total mass of active materials from both electrodes. Furthermore, the energy density of the assembled ASC device was calculated using the following equation.^54^

5 *E* (W h kg^−1^) = [(*C*_cell_ × Δ*V*^2^) × 0.5]/3.6

where *E* is the energy density (W h kg^−1^), *C* is the specific capacitance of the ASC (F g^−1^) and Δ*V* is the potential window (V). Also, the power density of the ASC was calculated using the following relationship:^53,54^

6 *P* (W kg^−1^) = 3600*E*/*t*_d_

where *P* is the power density and Δ*t* is the discharge time.

## 3. Results and discussion

### 3.1. Morphology characterization

**Fig. 2:**
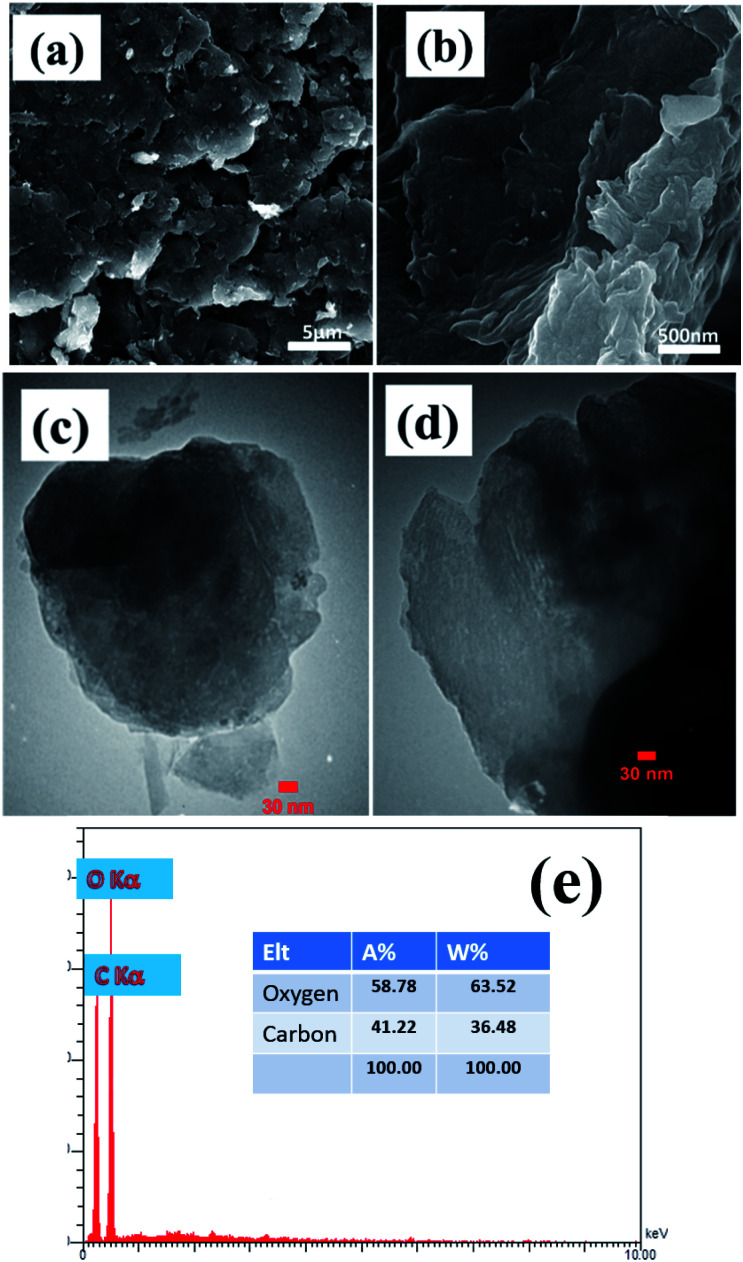
(a and b) FE-SEM and (c and d) TEM images and (e) EDS profile of the prepared porous graphene oxide.

The morphology and microstructure of the prepared powders and electrodes were studied *via* FE-SEM and TEM observations. FE-SEM images of the prepared graphene oxide are shown in Fig. 2. The low magnification FE-SEM image demonstrated sheet-like morphology of the synthesized graphene oxide (Fig. 2a). Fig. 2b shows a magnified FE-SEM image of the graphene oxide which reveals the presence of wrinkles on its surface that could increase its specific surface area. Fig. 2c and d show TEM images of the prepared graphene oxide sample. The TEM images show the completely porous texture of the synthesized graphene oxide along with some wrinkles, which suggests that the GO syntesized using our applied procedure has a thin porous structure. This morphology could result in the higher performance of the composite in charge storage tests. Fig. 2e shows the EDS elemental profile of the synthesized GO powder. It can be seen that carbon and oxygen are present in the composition of the GO at atomic percentages of 58.78% and 41.22%, respectively. The oxygen content of the GO sample indicates the successful oxidation of graphite using oxidizing agents, which results in exfoliation of the graphitic layers, primarily starting from the edge planes.

**Fig. 3:**
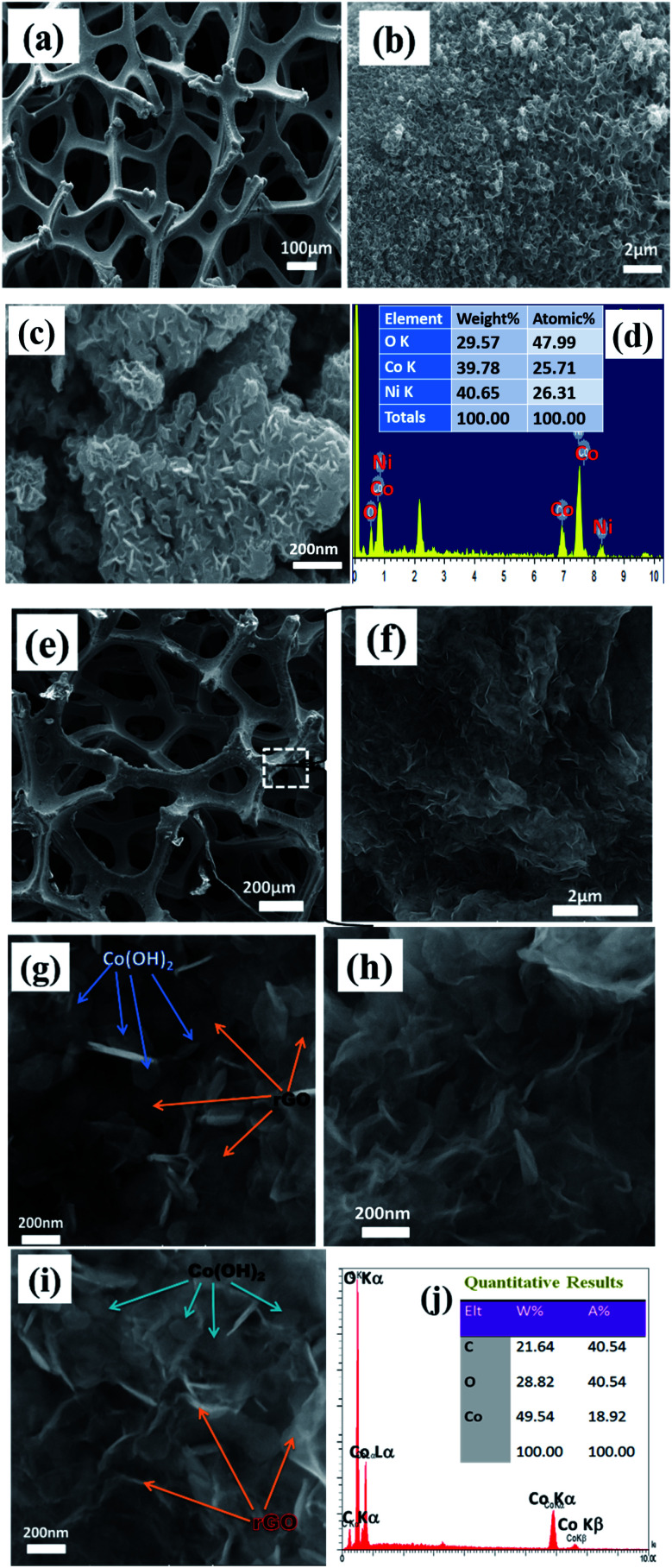
FE-SEM images and related EDS graphs of (a–d) Co(OH)_2_/Ni foam and (e–j) Co(OH)_2_@p-rGO/Ni foam.

After characterization of the GO sample, we aimed to characterize the fabricated Co(OH)_2_/NF and Co(OH)_2_@p-rGO/NF electrodes. Fig. 3a–d show FE-SEM images of the electrodeposited cobalt hydroxide along with its corresponding EDS spectrum as well as elemental percentage.

For the pristine hydroxide electrode, a uniform and thin film is observed in the low-magnification FE-SEM image (as seen in Fig. 3a). The high-magnification FE-SEM images of the electrodeposited cobalt hydroxide show the presence of nanosheets within the deposited material that are densely packed and nearly homogenously cover the entire surface of the Ni-foam support (Fig. 3b and c). And, the hierarchical nanosheets of cobalt hydroxide are obvious from Fig. 3c. Fig. 3d displays the EDS spectrum along with the percentage of each element present within the pristine Co(OH)_2_/NF electrode. The EDS spectrum verifies the presence of nickel, cobalt and oxygen in weight percentages of 40.6%, 39.8% and 29.6%, respectively. The presence of nickel originates from the Ni-foam support used for the electrodeposition of cobalt hydroxide and demonstrates the thin film deposition of cobalt hydroxide through applying a cathodic current. Fig. 3e–i show FE-SEM images and the EDS profile of the fabricated composite electrode. As obvious from the FE-SEM images, the surface morphology of the composite/NF electrode is quite different from that of the pristine cobalt hydroxide/NF electrode. The low-magnification FE-SEM image reveals the deposition of a uniform film onto the surface of the 3D Ni-foam support (Fig. 3e). From Fig. 3f, it is clearly observable that the deposited thin film is composed entirely of a sheet-like morphology. The high-magnification FE-SEM images indicates that the presence of the GO within the composite structure does not change the precipitation of cobalt hydroxide nanosheets on the surface of the foam substrate. However, it seems that in the presence of electrochemical p-rGO the nanosheets show a thinner morphology. The location of each component, including cobalt hydroxide and reduced GO, are shown in Fig. 3g and h, where the excellent dispersion of the cobalt hydroxide plates within the reduced GO sheets can be observed. All the observed Co(OH)_2_ plates have well-defined hexagonal shapes at the nanoscale. Fig. 3j exhibits the EDS spectrum of the composite electrode. The EDS data shows the presence of Co, C and O in atomic percentages of 18.9%, 40.5% and 40.5%. The absence of nickel within the studied area demonstrates the full coverage of the Ni-electrode surface by a film of the composite material.

**Fig. 4:**
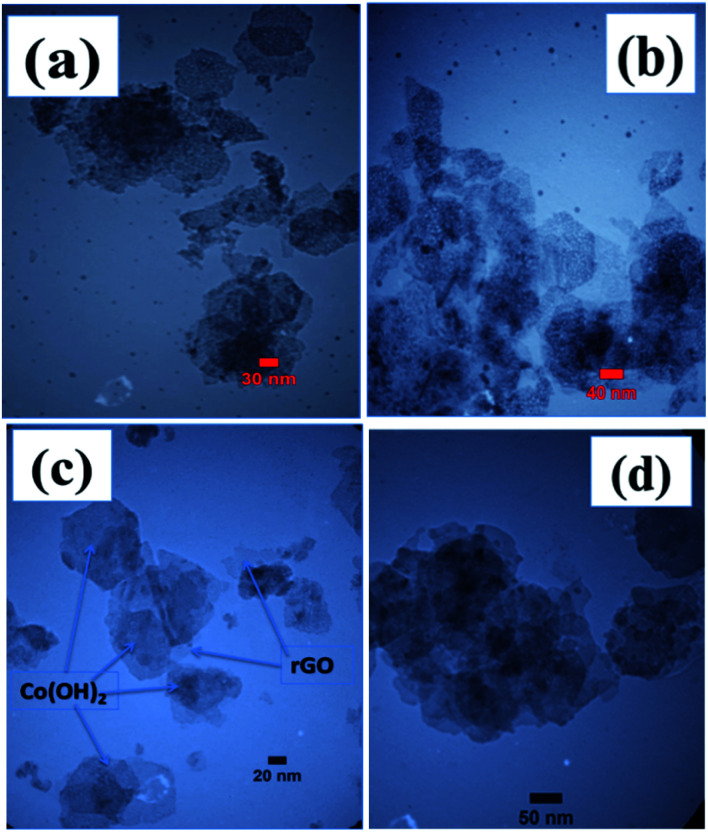
TEM images of the fabricated (a and b) Co(OH)_2_ and (c and d) Co(OH)_2_/p-rGO.

**Fig. 5:**
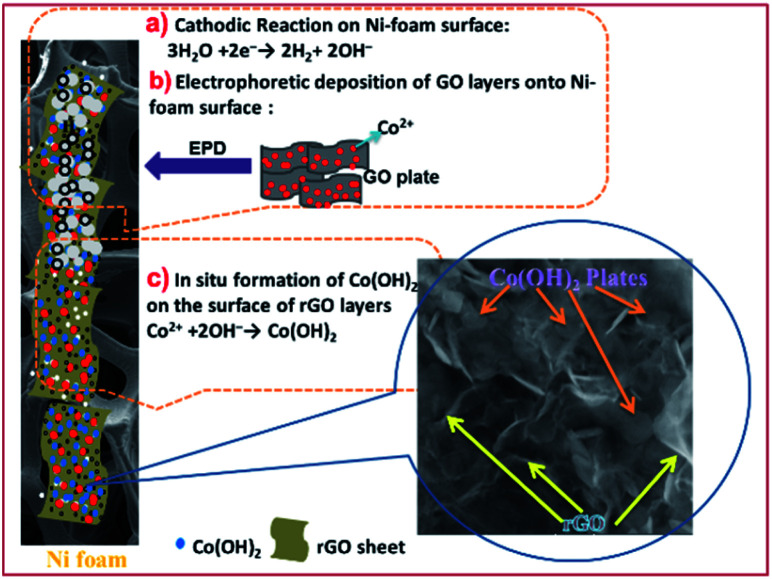
Mechanisms of the electrophoretic/electrochemical deposition processes: (a) base generation step, (b) electrophoretic deposition of rGO onto NF and (c) the chemical formation of Co(OH)_2_ on rGO.

Fig. 4a–d present the TEM images of the fabricated samples scrubbed from the surface of the nickel sheet electrode. Overall, all of the samples show a plate-like morphology with a relatively hexagonal shape. However, the electrochemically reduced GO within the composite material has a thinner morphology in comparison to the cobalt hydroxide nanoplates. The cobalt hydroxide plates have a thickness in the order of several nanometers, as revealed in the TEM images of both samples. For the prepared pristine hydroxide, hexagonal plates with a completely porous texture can be observed in the TEM images (Fig. 4a and b). Surprisingly, the observed plates have uniform pores, which may be due to the fast H_2_ bubbling resulting in a large amount of H_2_ being releasing on the formed hydroxide plates on the cathode surface during the applied high current deposition conditions. In the composite (Fig. 4c and d), the hydroxide plates are observed on the GO sheets, revealing their *in situ* electrochemical growth after the electrophoretic deposition of GO nanosheets on the cathode surface (as shown in Fig. 5). It seems that the presence of rGO reduces the aggregation of the cobalt hydroxide nanoplates (Fig. 4c and d). Like for the pristine sample, the hydroxide plates grown on the GO sheets have porous morphology, as can be seen in Fig. 4c. Moreover, it seems that the cobalt hydroxide nanoplates are mostly regularly distributed in the reduced GO matrix to produce the composite material (Fig. 4d). The mechanisms of the electrophoretic deposition of rGO and *in situ* electrochemical growth of the Co(OH)_2_ plates are shown in Fig. 5. In the electrophoretic deposition (EPD) of rGO, two sub-processes take place: (i) the electrophoretic movement of suspended charged particles in a liquid phase towards the electrode under the effect of an electric field; (ii) collection of the particles at the electrode surface and formation of a deposit.^51^ In our case, reduction of water molecules first occurs on the Ni foam (as shown in step a in Fig. 5) and the pH of the Ni foam surface increases. Then, the GO plates move towards the cathode side and are deposited on the Ni foam surface (step b in Fig. 5). Finally, Co^2+^ cations are chemically reacted with the OH^−^ ions to form Co(OH)_2_ on the rGO sheets (step c in Fig. 5).

### 3.2. Structural characterization

**Fig. 6:**
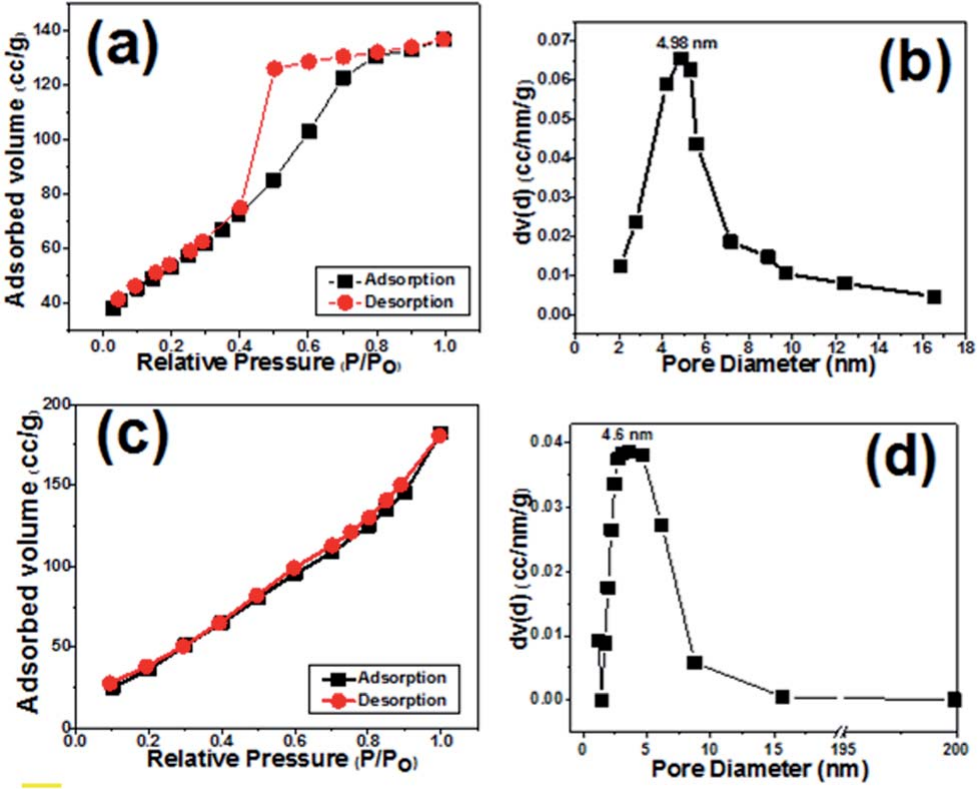
N_2_ adsorption/desorption isotherms and pore size distributions of (a and b) the prepared cobalt hydroxide and (c and d) the Co(OH)_2_@porous rGO composite.

Surface area and pore size distribution of the prepared samples were investigated *via* Brunauer–Emmett–Teller (BET) gas-sorption measurements. Fig. 6a presents the nitrogen adsorption/desorption isotherm of the cobalt hydroxide nanoplates, and Fig. 6b displays the corresponding Barrett–Joyner–Halenda (BJH) pore size distribution curve. The BET curve of the prepared porous nanoplates is a type III hysteresis loop isotherm. The surface area of Co(OH)_2_ sample was measured to be 149.56 m^2^ g^−1^, with a mean pore diameter of 4.98 nm (Fig. 6a and b), demonstrating the high specific surface area of the cobalt hydroxide nanoplates along with its mesoporous texture.

For the composite sample, the BET profile also shows a type IV hysteresis loop, and the surface area of the hybrid material was found to be 197.2 m^2^ g^−1^ (Fig. 6c and d). As obvious from Fig. 6d, the composite material has a major pore size of 4.6 nm, demonstrating the presence of mesopores within this material. These observations imply the presence of several voids among the Co(OH)_2_ and p-rGO nanosheets. The porous morphology of both samples could be suitable for diffusion of alkaline ions (OH^−^) within the fabricated electrodes. Thus, it is expected that the fabricated composite could provide higher charge storage capability due to its higher surface area when compared to the pristine electrode.

**Fig. 7:**
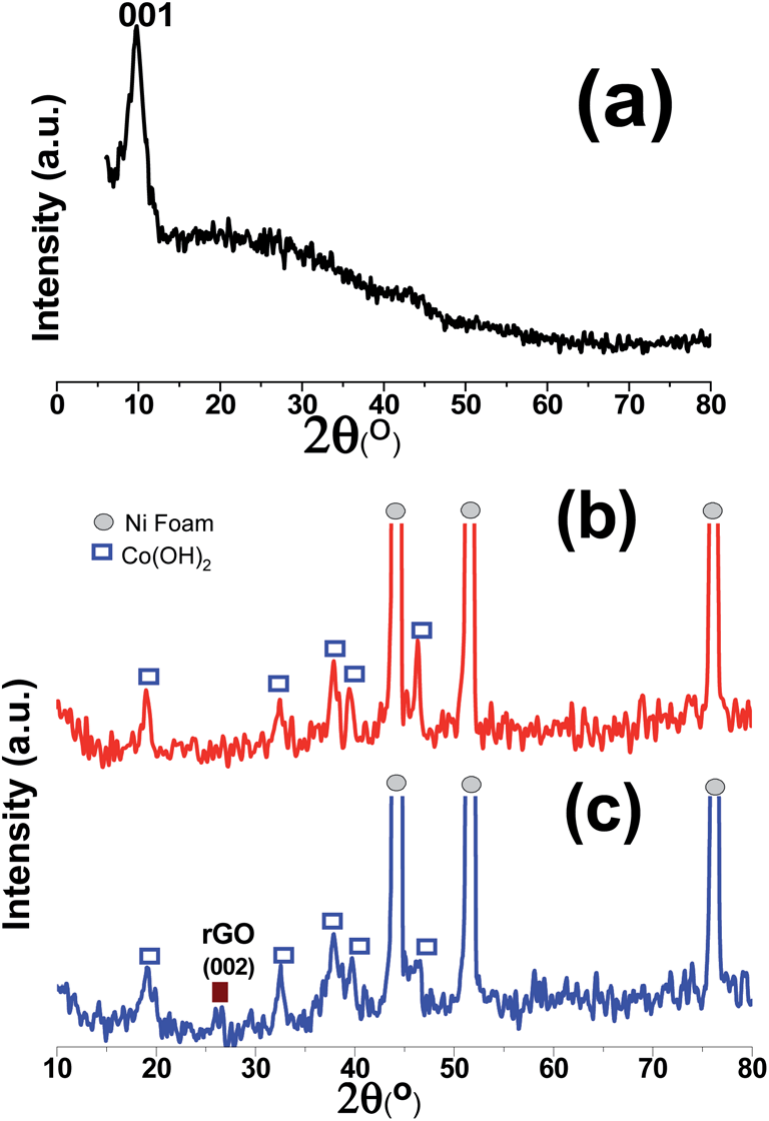
XRD patterns of (a) the prepared graphene oxide powder, and the fabricated (b) Co(OH)_2_/Ni foam and (c) Co(OH)_2_@p-rGO/Ni foam electrodes.

The XRD patterns of the GO powder, Co(OH)_2_/NF and Co(OH)_2_@p-rGO/NF electrodes are displayed in Fig. 7. The as-prepared GO shows a very intense diffraction peak at 2*θ* = 9.89° with a d-spacing of 0.925 nm (Fig. 7a), which corresponds to the (001) plane,^35,37^ hence confirming the synthesis of GO *via* the modified Hummers' method and indicating the exfoliation of the graphite sheets *via* the applied route. In the XRD pattern of Co(OH)_2_/NF (Fig. 7b), there are three sharp diffraction peaks, which can be ascribed to the (111), (200) and (220) crystal planes of the Ni foam substrate. Moreover, several diffraction bands relating to (001), (100), (101), (102), (110), (111), (103), and (201) can be clearly observed in Fig. 7b, with all of these peaks being in good line with β-Co(OH)_2_ according to the standard card (ICDD 30-0443).^44^ In the case of the Co(OH)_2_@p-rGO/NF electrode (Fig. 7c), all of the mentioned diffraction peaks for cobalt hydroxide can be easily observed and an one extra diffraction peak is located at 2*θ* = 25.89°, which is related to the p-rGO sheets. The low intensity of the diffraction peak related to p-rGO in the XRD pattern of the composite electrode is probably due to the more disordered stacking and almost homogenous distribution of the rGO sheets in the fabricated Co(OH)_2_@p-rGO/NF electrode.^35,37^

**Fig. 8:**
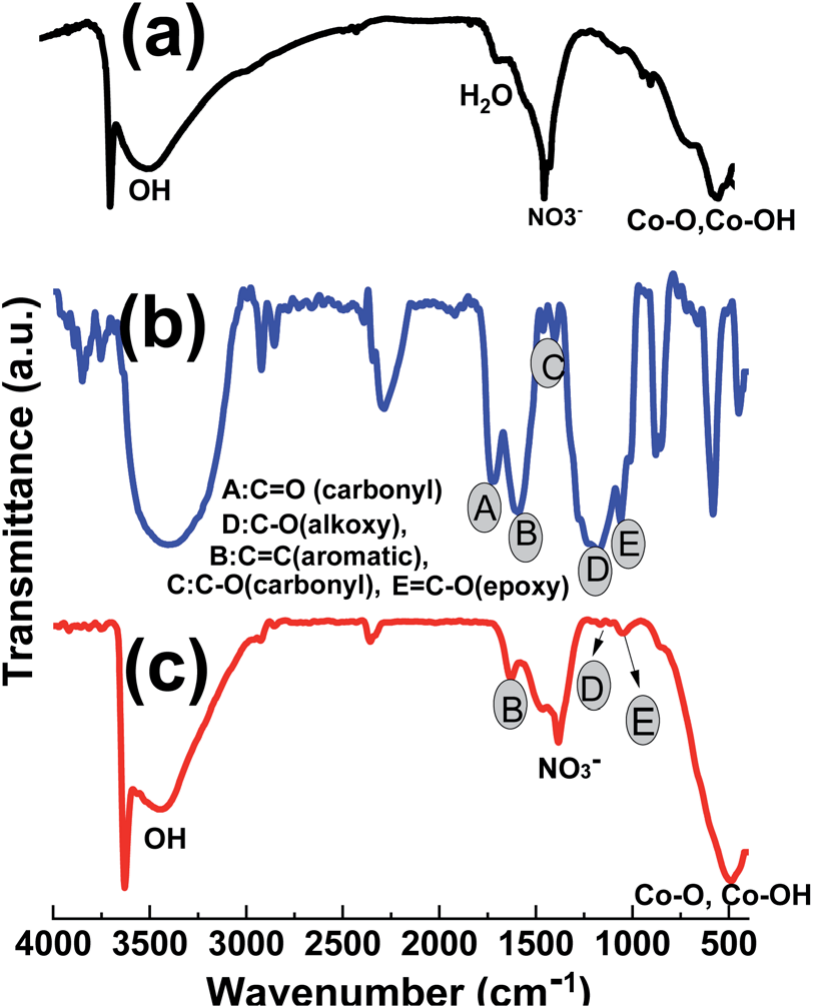
IR spectra of the prepared (a) GO powder, (b) pristine Co(OH)_2_ and (c) Co(OH)_2_@p-rGO composite.

The FTIR spectra of the prepared GO powder, pristine Co(OH)_2_ and Co(OH)_2_@p-rGO composite are given in Fig. 8. For the hydroxide sample in Fig. 8a, a shoulder band at 3641 cm^−1^ is ascribed to the non-hydrogen attached hydroxyl moieties in brucite-like β-Co(OH)_2_.^52^ The peaks at wavenumbers of 491 cm^−1^ and 522 cm^−1^ can be attributed to the Co–O stretching modes and Co–OH bending modes of Co(OH)_2_.^53^ These data imply Co(OH)_2_ precipitation on the cathode electrode. The wide peak at 3450 cm^−1^ is related to the vibration of hydrogen-bonded OH moieties in the inter-lamellar area of Co(OH)_2_.^53,54^ Also, the peak at around 1400 cm^−1^ may be due to the intercalated NO_3_^−^ ions within the layered structure of the cobalt hydroxide nanoplates. In the case of GO, the IR spectrum has several characteristic absorption peaks related to GO (Fig. 7b), which are specified with A–E letters in Fig. 6b; C=O stretching vibration at 1744 cm^−1^, the O–H stretching at 3425 cm^−1^ and 1393 cm^−1^, aromatic C=C stretching vibration at 1626 cm^−1^, epoxy C–O stretching vibration at 1221 cm^−1^ and the alkoxy C–O stretching vibration at 1057 cm^−1^.^53–56^ For the composite sample, as seen in Fig. 8c, all the IR peaks related to cobalt hydroxide are present, which are: the sharp band at 3643 cm^−1^ attributed to the non-hydrogen bonded hydroxyl moiety in brucite-like β-Co(OH)_2_, and Co–O stretching modes and Co–OH bending vibrations at 526 cm^−1^ and 497 cm^−1^, respectively. The wide peak at 3450 cm^−1^ corresponds to the stretching mode of hydrogen-bonded OH moieties within the cobalt hydroxide material. Also, the peak at around 1400 cm^−1^ can be ascribed to the intercalated NO_3_^−^ ions within the layered structure of the cobalt hydroxide nanoplates.^57^ It can also be seen that the intensity of the absorption peaks of oxygen-containing functionalities like alkoxy (1062 cm^−1^) and epoxy (1225 cm^−1^) groups is significantly reduced. The carbonyl C=O and carboxyl peaks are also not present in the IR spectrum of the composite material (Fig. 8c), indicating the reduction of GO during the electrodeposition. However, there is not a decrease in the intensity of all peaks for oxygen-containing moieties, some are left, implying that GO is not completely reduced to graphene *via* the electrochemical method but that this process only occurs partially. These oxygen containing moieties could be helpful for better interactions among the cobalt hydroxide plates and partially reducing the GO components.

**Fig. 9:**
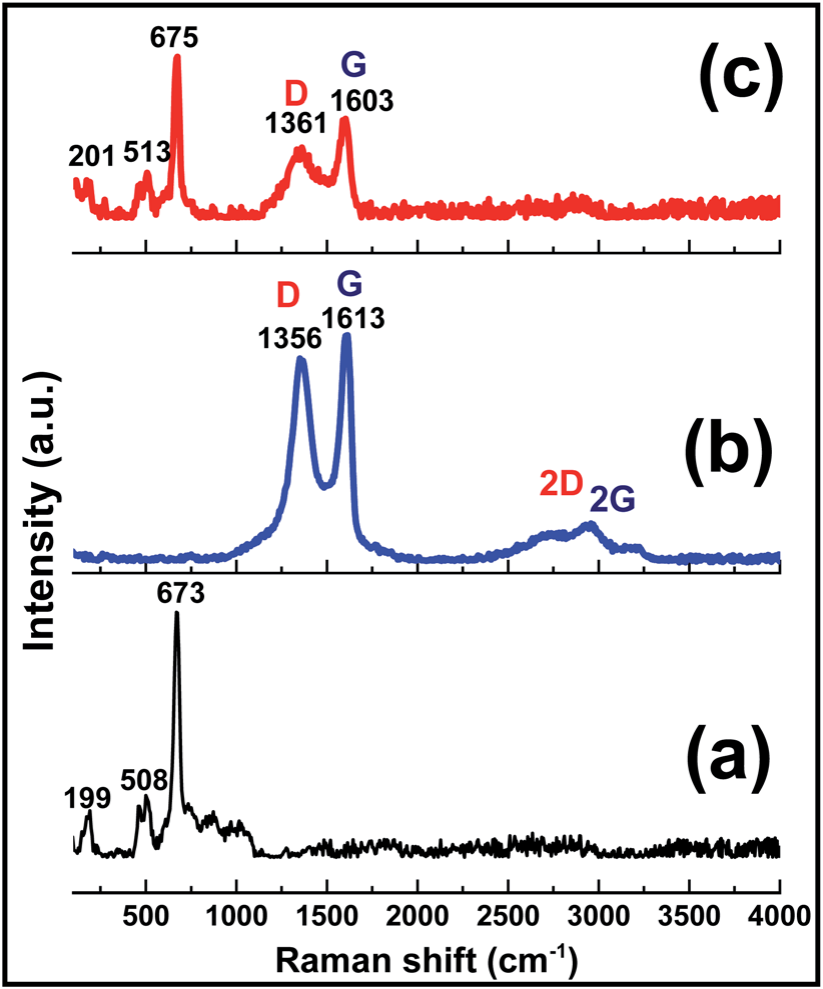
Raman spectra of the prepared (a) pristine Co(OH)_2_, (b) GO powder and (c) Co(OH)_2_@p-rGO composite.

The composition and structural features of the prepared GO, Co(OH)_2_ and Co(OH)_2_@p-rGO samples were also characterized through Raman spectroscopy analysis (Fig. 9). The pure Co(OH)_2_ spectrum shows two major peaks at 508 and 673 cm^−1^ (Fig. 9a). These peaks are due to the Eg and A_1g_ modes of the cobalt hydroxide material.^58^ The GO sample (Fig. 9b) exhibits three peaks at 1351, 1613, 2783 and 2943 cm^−1^, which are well known as D, G, 2D and 2 G bands, respectively. The G band is related to the vibration of graphitic sp^2^ carbons and in fact reflects graphitic domains, while the D peak is attributed to sp^3^ carbon atoms and reflects the defects correlated to the vacancies and grain boundaries within carbonaceous materials. Another band positioned at around 2783 cm^−1^ can be ascribed to a two phonon inter-valley double resonance mechanism. Rather than the mentioned bands, the bands at about 2970 and 3189 cm^−1^ can be ascribed to the D + D′ and 2D′ peaks, with these peaks being related to intra-valley scattering due to lattice defects and the overtone of the phonon modes that give rise to the D′ bands, respectively.^59–61^ It is known that the *I*_D_/*I*_G_ ratio reflects the degree of defects present in the GO sample, which was as high as 0.9.^59^ Compared with the pristine GO sample, the composite sample exhibits the characteristic peaks of both the Co(OH)_2_ and rGO materials, which confirm its Co(OH)_2_@rGO composition. The Raman shifting of the cobalt hydroxide peaks to higher values could be ascribed to the possible interaction between cobalt hydroxide and the rGO material.^42^ Furthermore, in good agreement with the XRD analysis, the Raman results also provide evidence of the surface coverage of the rGO with cobalt hydroxide nanoplates (Fig. 9c). The presence of rGO in the material is anticipated to improve its electrochemical effectiveness.

**Fig. 10:**
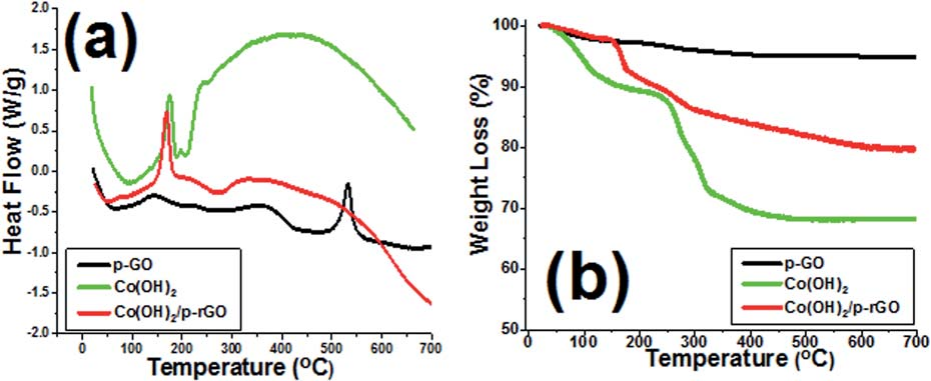
TGA/DSC profiles of the prepared GO powder (p-GO), pristine Co(OH)_2_ and Co(OH)_2_@p-rGO composite.

The TGA as well as DSC analyses of Co(OH)_2_ and p-GO and Co(OH)_2_@p-rGO are presented in Fig. 10. The synthesized porous GO sample shows a broad endothermic peak in its DSC curve (Fig. 10a) and correspondingly a weight loss at around 100 °C due to the evaporation of water molecules adsorbed on the surface sample (Fig. 10b). Another weight loss between 200 and 400 °C is related to the decomposition of labile functional moieties within the sample (Fig. 10b). This change is observed in the DSC profile as two successive endothermic peaks (Fig. 10a). Another mass loss in the range of 400–600 °C accompanied by an exothermic DSC peak at 550 °C is thought to be related to the decomposition of GO.^63^ The cobalt hydroxide sample shows a major weight loss centred at around 180 °C, which is due to the conversion of cobalt hydroxide to cobalt oxide (Co_3_O_4_).^57,64^ In the case of the composite material, the similar weight loss at below 150 °C is also related to the removal of adsorbed water molecules as well as crystalized water molecules from the sample. The weight loss at around 185 °C is attributed to the conversion of cobalt hydroxide to Co_3_O_4_, as reported previously in literature.^64,65^ Total weight losses of 5.18%, 31.81% and 20.75% are observed for the GO powder, pristine Co(OH)_2_ powder and their composite, respectively (as seen in Fig. 10b). The lower weight loss of the composite material can be attributed to the lower loading of this material within the composite sample as well as the presence of rGO.

### 3.3. Electrochemical characterization

#### 3.3.1. Three-electrode system

**Fig. 11:**
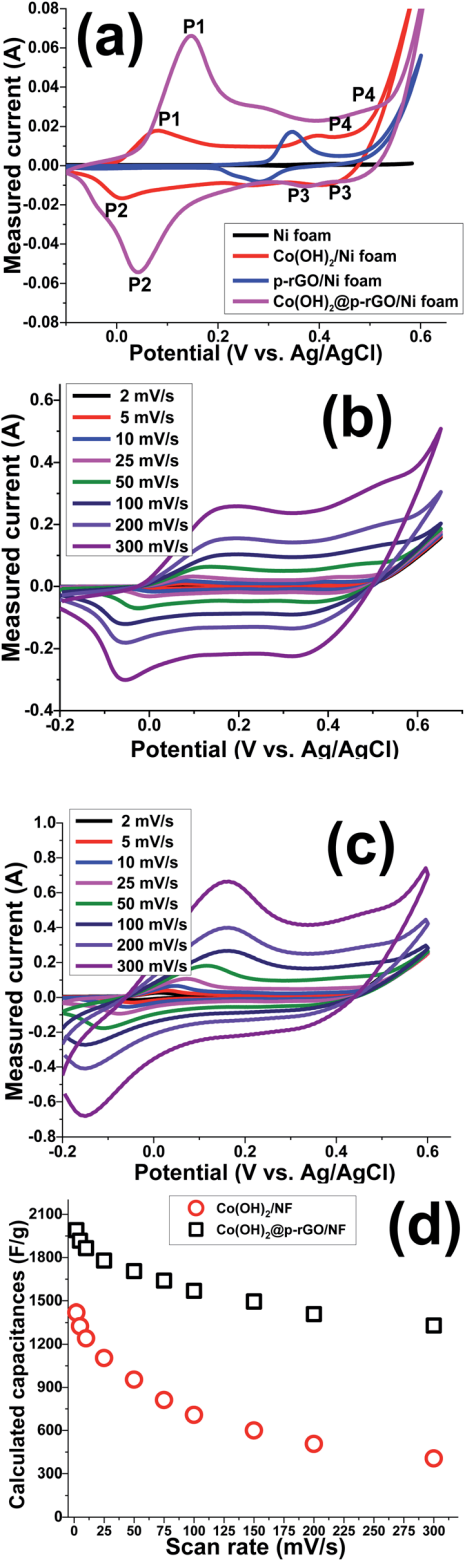
(a) CVs of the fabricated electrodes (*i.e.* blank NF, Co(OH)_2_/NF and composite/NF) at 10 mV s^−1^, CV plots of the (b) Co(OH)_2_/NF and (c) composite/NF electrodes at the various scan rates, and (d) the calculated specific capacitances *vs.* the scan rate.

To evaluate the electrochemical characteristics of the fabricated electrodes, CV, GCD and EIS tests were conducted in an aqueous solution of 2 M KOH. Fig. 11a shows the CV curves of the bare nickel foam support, Co(OH)_2_/NF, p-rGO/NF and Co(OH)_2_@p-rGO/NF electrodes at a scan rate of 10 mV s^−1^ within a potential range of −0.2 to 0.6 V. The nickel foam substrate shows minimum enclosed area under its CV curves and hence negligible pseudocapacitance contribution in the capacitance of the fabricated Co(OH)_2_/NF and Co(OH)_2_@p-rGO/NF electrodes. Moreover, the enclosed area under the CV curve of Co(OH)_2_ was significantly increased after the deposition of cobalt hydroxide onto Ni foam (Fig. 11a). In the case of the composite electrode, a similar current–potential was observed and the enclosed area under the CV curve is surprisingly increased, with two sharp redox peaks exhibited (Fig. 11a). Both the CVs of the Co(OH)_2_/NF and Co(OH)_2_@p-rGO/NF electrodes show redox couples relating to the conversion of Co(ii) to Co(iii) and Co(iii) to Co(iv) during anodic scanning, with these redox reactions being reversed during cathodic scanning:^52,57^

7 Co(OH)_2_ + OH^−^ ⇌ CoOOH + H_2_O + e^–^ (*p*_1_/*p*_2_)

8 CoOOH + OH^−^ ⇌ CoO_2_ +H_2_O + e^–^ (*p*_3_/*p*_4_)

Hence, it can be said that the capacitances of the fabricated electrodes mainly originate from pseudocapacitance behavior rather than EDLC due to presence of fast redox reactions at the interface of the Co(OH)_2_ platelets/alkaline electrolyte. The rGO/NF electrode shows higher specific capacitance when compared to the NF blank foam, which is due to the EDL capacitive behavior of rGO. The porous rGO layers increase the diffusion of electrolyte ions to the interface of the electrolyte/electrode and improve the capacitance of the material. Introduction of the porous rGO nanosheets to the Co(OH)_2_ active material resulted in the further enhancement of the enclosed area under the CV curves (as seen in Fig. 11a), indicating the better electrochemical performance of the nanocomposite electrode in comparison to the single analogous electrodes *i.e.* Co(OH)_2_/NF and p-rGO/NF.

Fig. 11b and c show CV plots of the Co(OH)_2_/Ni foam and Co(OH)_2_/p-rGO/Ni foam electrodes at different scan rates of 2, 5, 10, 25, 50, 100, 200 and 300 mV s^−1^. It can be seen that the shape of the CV plots does not alter significantly, demonstrating the reversibility of the electrochemical behaviour of the fabricated Co(OH)_2_ electrodes within the potential range and applied scan rates.^45–47^ Shifts of redox materials in the positive and negative directions correspond to the quasi-reversible nature of faradaic reactions that take place at the interface of the electrolyte/electrode as well as the polarization of the electrodes. In addition, the CV curves of Co(OH)_2_@rGO/NF (Fig. 11c) have a different shape compared to those of the pristine cobalt hydroxide electrode, which is due to the presence of rGO that has a high specific surface area that promotes EDLC behaviour. This means that p-rGO induces an increase in the electrochemical activity of the Co(OH)_2_ composite electrode compared with that of the pure hydroxide/Ni foam electrode (Fig. 11b).

The Co(OH)_2_@p-rGO/NF hybrid electrode showed higher specific capacitances than the Co(OH)_2_/NF electrode at all scan rates, which, as previously mentioned, demonstrates the better electrochemical performance of the composite electrode. The highest specific capacitance of 1990 F g^−1^ was attained for the composite electrode at a scan rate of 2 mV s^−1^ which is much higher than that of the Co(OH)_2_ electrode (1420 F g^−1^). This improvement highlights the supporting role of the porous GO layers in the capacitance performance of the fabricated hybrid electrode (as is obvious from Fig. 11a). The calculations show that the specific capacitances of the composite electrode are 1990, 1916, 1864, 1778, 1703, 1638, 1566, 1496, 1409 and 1329 F g^−1^ at scan rates of 2, 5, 10, 20, 50, 75, 100, 150, 200 and 300 mV s^−1^, respectively (as presented in Fig. 11d). It was calculated that the Co(OH)_2_/NF electrode also delivers specific capacitance values of 1420, 1324, 1241, 1104, 953, 812, 708, 601, 507, and 406 F g^−1^ at scan rates of 2, 5, 10, 20, 50, 75, 100, 150, 200 and 300 mV s^−1^, respectively (Fig. 11d). These data indicate that the Co(OH)_2_/p-rGO composite electrode retains more than 65% of its primary capacitance upon increasing the scan rate to more than 150 times, indicating the high-rate capability of the composite electrode, and a performance that is better than that of the Co(OH)_2_/Ni electrode (*i.e.* 28.5% capacitance retention over scan rates of 2 to 300 mV s^−1^).

**Fig. 12:**
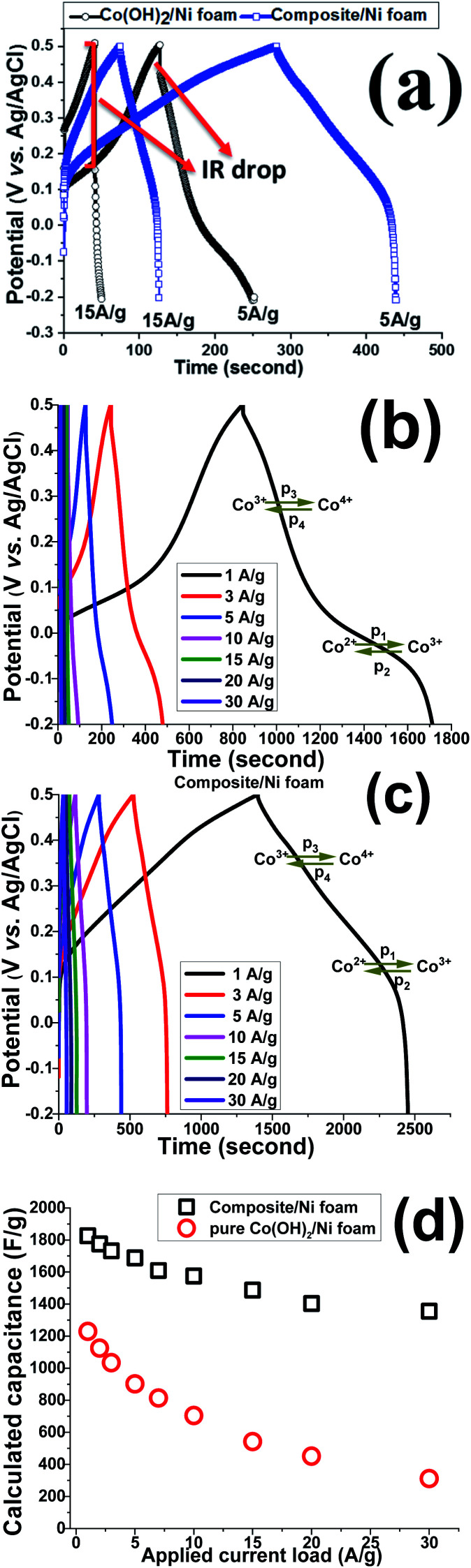
(a) GCD profiles of the fabricated electrodes at currents of 5 A g^−1^ and 15 A g^−1^, (b and c) GCD curves of Co(OH)_2_/NF and composite/NF electrodes at different current loads, and (d) calculated capacitances at various loads of 1 to 30 A g^−1^.

GCD experiments were carried out to further elucidate the capacitance behavior of both active materials. Fig. 12a shows the GCD curves of the pristine and nanocomposite electrodes at current densities of 5 and 15 A g^−1^. In a similar trend to the CV results, pseudo-capacitance behavior was observed as the major capacitive behavior in the GCD plots of the pristine hydroxide electrode (*i.e.* Co(OH)_2_/Ni foam), with EDLC capacitor behavior being absent (Fig. 12a).

In fact, for this electrode, two major potential steps are observed in its GCD curves, which correspond to Co^2+^/Co^3+^ and Co^3+^/Co^4+^ redox faradaic reactions. In contrast, both EDL and faradaic behaviors are observable for the Co(OH)_2_@p-rGO/NF electrode (Fig. 12a), which originate from the p-rGO and cobalt hydroxide components of the fabricated composite, respectively. Furthermore, the IR drop for the composite electrode is much lower than that of the pristine electrode (as clearly denoted in Fig. 12a). It is also obvious that by increasing the current load, the IR drop increased for the pristine Co(OH)_2_/NF electrode, which is due to the high contact resistances within the electrode/electrolyte interface as well as the low electrical conductivity of the hydroxide nanoplates. However, the IR drops are negligible in the case of the Co(OH)_2_@rGO/NF electrode, indicating the better redox performance of the cobalt hydroxide in the presence of the conductive rGO network. Fig. 12b and c show the GCD plots of the Co(OH)_2_/NF and Co(OH)_2_@p-rGO/NF electrodes at various current loads from 1 to 30 A g^–1^. The shapes of the GCD profiles of the pristine hydroxide indicate its pure pseudocapacitive behavior (Fig. 12b), while the hybrid behavior of EDL and pseudocapacitance is observed for the composite electrode (Fig. 12c). And, it can be clearly observed that the capacity retention is better in the composite profile upon increasing the applied current (Fig. 12d).

Using eqn (2), the capacitance values of the fabricated electrodes were calculated and are shown in Fig. 12d. The specific capacitances for the Co(OH)_2_/Ni foam electrode were respectively calculated at current loads of 1, 2, 3, 5, 7, 10, 15, 20 and 30 A g^−1^ to be as high as 1229, 1125, 1034, 902, 814, 705, 542, 451 and 311 F g^−1^, where the composite/Ni foam electrode was calculated to be able to deliver capacitance values of as high as 1826, 1775, 1732, 1688, 1611, 1575, 1487, 1403 and 1355 F g^−1^ at similar current densities. Furthermore, the pristine and composite electrodes exhibit 37% and 75.5% capacity losses with an increase in the applied current loads from 1 to 30 A g^−1^. These data prove the better capacitance of the composite electrode compared with that of the pristine hydroxide electrode. The reason for the decreasing charge storage capacity of the electrodes is due to at low current densities nearly all of the active sites within the electrode being accessible for electrolyte ion diffusion during the course of the time of the electrochemical testing; thus, by increasing the current density, the length of time of the experiment was decreased due to the inaccessibility of some parts of the electrode for electrolyte ion diffusion. In other words, some parts of the electrode are not available for electrolyte ion diffusion at a high current density, which results in specific capacitance fading. However, these results demonstrate the better performance of the composite material due to the good contact between the two components within the composite material as well as the high specific interfacial area between the two components.

Based on the capacitance values and discharge times of the fabricated Co(OH)_2_/NF and Co(OH)_2_@p-rGO/NF electrodes, an energy density of 83.5 W h/kg and power density of 0.321 kW kg^−1^ (for the pristine hydroxide/NF electrode) and energy density of 124 W h kg^−1^ and power density of 0.424 kW kg^−1^ (for the composite electrode) were achieved.

**Fig. 13:**
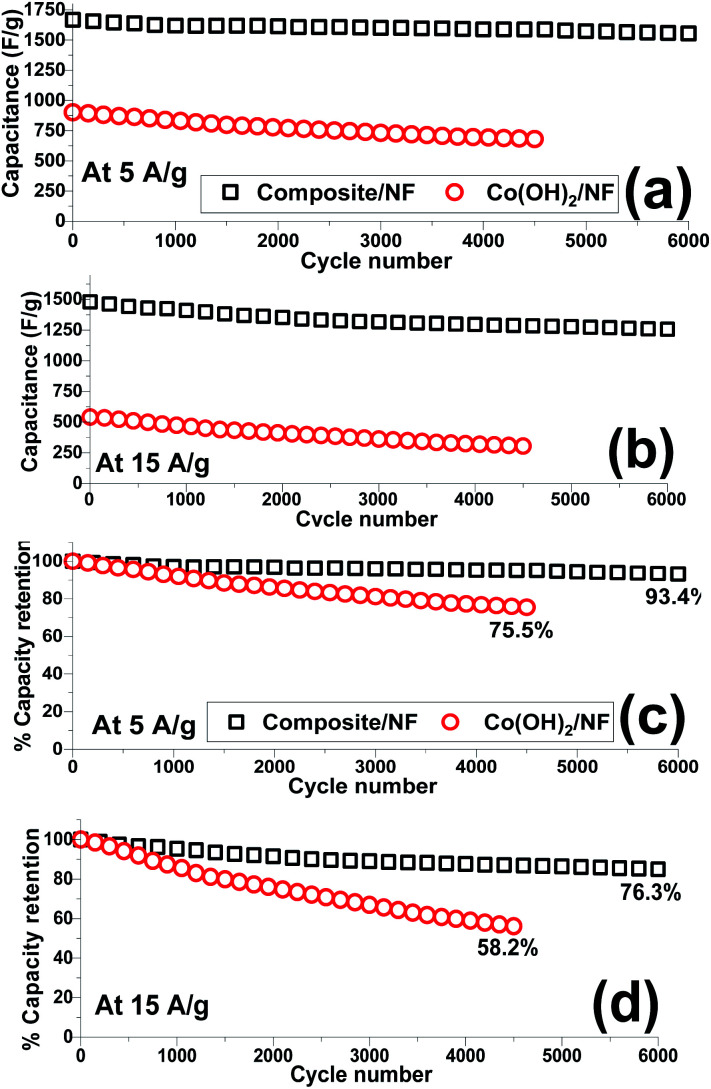
Cycling data of the Co(OH)_2_/Ni and composite/Ni electrodes at current loads of (a and c) 5 A g^−1^ and (b and d) 15 A g^−1^.

The capacity retention of the electroactive material is an important parameter by which to evaluate its ability as an electrode material. Hence, the cycling data of the fabricated Co(OH)_2_/Ni and composite/Ni electrodes were recorded in 2 M KOH electrolyte through continuous 4500 and 6000 charge–discharge cycles at current densities of 5 and 15 A g^−1^, respectively. In this regard, the capacitance of each cycle (Fig. 13a and b) together with its reduction in comparison to the previous cycle (Fig. 13c and d) were calculated. The obtained specific capacitances and capacity losses are shown in Fig. 13. Overall, it was found that the composite electrode is capable of being cycled for up to 6000 cycles without any substantial reduction in the delivered capacitances even at a very high discharge current density of 15 A g^−1^. In contrast, a significant decrease was observed in the calculated capacitances, especially at a high current load of 15 A g^−1^, when the hydroxide/NF electrode was cycled for 4500 consecutive cycles. It can be seen that the hybrid electrode material exhibits good cycling stabilities of 93.4% and 78.3% after 6000 cycles at discharge currents of 5 and 15 A g^−1^, respectively (Fig. 13c and d). In fact, it was observed that the composite electrode can still deliver capacitance values of 1555 F g^−1^ and 1254 F g^−1^ after the 6000th cycle at current densities of 5 A g^−1^ and 15 A g^−1^, respectively (Fig. 13a and b). However, the Co(OH)_2_/Ni electrode shows relative poor cycling abilities of 75.5% and 58.2% after 4500 cycles at discharge currents of 5 and 15 A g^−1^, respectively (Fig. 13c and d), and a decrease in its capacity values from 902 to 681 F g^−1^ (at 5 A g^−1^) and from 542 to 304 F g^−1^ (at 15 A g^−1^) after the end of the 4500th GCD cycle. These obtained results verify the good capacitive behavior of the Co(OH)_2_@porous reduced GO composite material and further reveal the synergy effect between the rGO and cobalt hydroxide.

A comparison of the electrochemical performances of some cobalt-based composite electrodes is shown in Table 1. It can be observed that the specific capacitance, cyclability and high-rate performance of our electrode are comparable with those reported in the literature. Hence, this proves that the fabricated Co(OH)_2_/p-rGO/NF electrode exhibits high specific capacitance, long cycling life and outstanding rate capability. The reasons for such an improvement and high electrochemical performance of Co(OH)_2_@p-rGO/NF can be explained on the basis of the presence of pores, high specific surface area and electrical conductivity of the rGO sheets within the composite structure.

**Table 1:** Comparison of the electrochemical performances of various cobalt-based composite electrodes

Electrode material	Specific capacitance (F g^−1^)	Cycling stability (%)	Energy density (W h kg^−1^)	Power density (kW kg^−1^)	Ref
CC@CoMoO_4_–Co(OH)_2_	2020 at 1 A g^−1^	94.5 after 5000 cycles at 8A g^−1^	61	0.8	66
CoAl LDH/RGO	1296 at 1 A g^−1^	90.5 after 1000 cycles at 10 A g^−1^	—	—	67
Co(OH)_2_/graphene/NF	693.8 at 2 A g^−1^	91.9% after 3000 cycles at 40 A g^−1^	—	—	68
GO/Co–Mn composite	1231 at 3 A g^−1^	95% after 2000 cycles at 5 A g^−1^	—	—	69
CoMn LDH/CF	1079 at 2 A g^−1^	8.3% after 6000 cycles at 2 A g^−1^	—	—	70
CuCo_2_S_4_@Co(OH)_2_	2066 at 1 A g^−1^	84.5% after 5000 cycles at 10 A g^−1^	37.6	0.795	71
3D Co(OH)_2_/CNF	1186 at 1 A g^−1^	94.9% after 5000 cycles at 1 A g^−1^	—	—	72
β-Co(OH)_2_	1066 at 2 A g^−1^	86% after 5000 cycles at 5 A g^−1^	—	—	73
α-Co(OH)_2_/Ni	613 at 2 A g^−1^	70% after 5000 cycles at 5 A g^−1^	—	—	73
Ni–Co–S/Co(OH)_2_	1560 at 1 A g^−1^	81.7% after 2000 cycles at 10A g^−1^	—	—	74
Co(OH)_2_/NF	1227 at 1 A g^−1^	75.5% after 4500 cycles at 5 A g^−1^	83.5	0.321	This work
		58.2 after 4500 cycles at 15 A g^−1^	—	—	
	902 at 5 A g^−1^		61.3	0.183	
Co(OH)_2_@p-rGO/NF	1826 at 1 A g^−1^	93.4% after 6000 cycles at 5 A g^−1^	124	0.424	This work
	1688 at 5 A g^−1^	76.3% after 6000 cycles at 15 A g^−1^	114.8	0.365	

**Fig. 14:**
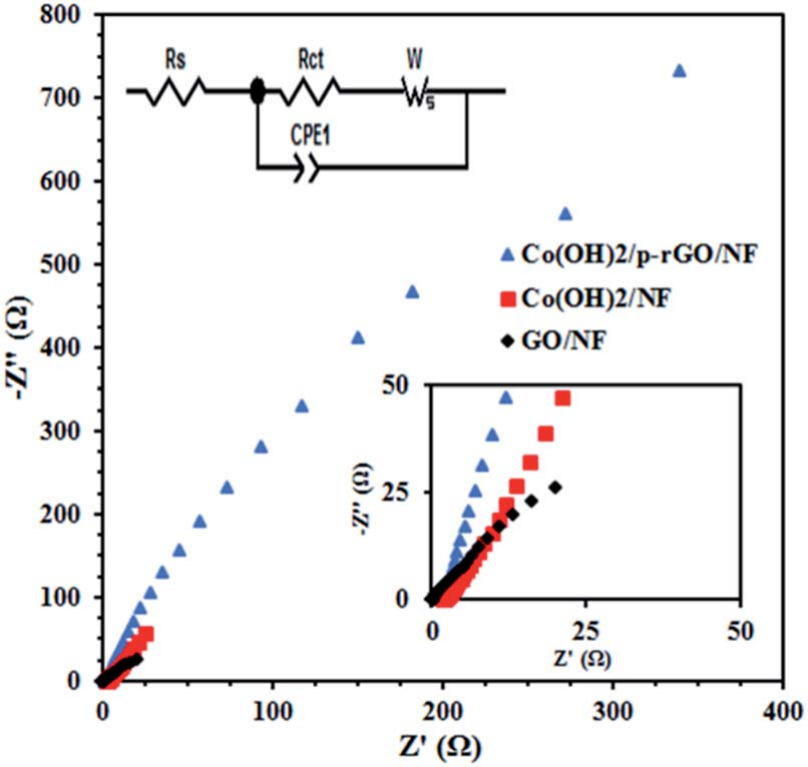
Nyquist plots of the GO/NF, Co(OH)_2_/NF and Co(OH)_2_/p-rGO/NF electrodes at the open circuit potential over a frequency range of 100 kHz to 0.01 Hz. The insets show both the high frequency region and corresponding equivalent circuit.

Nyquist plots of the composite, cobalt hydroxide/NF and GO/NF electrodes in the frequency range of 100 kHz and 10 mHz are shown in Fig. 14. These Nyquist plots correspond to an equivalent circuit comprising solution resistance, charge transfer resistance and constant phase and Warburg elements. *R*_s_ is commonly used to represent electrolyte resistance but also includes other uncompensated resistances determined from the intersection of the Nyquist plot with the horizontal axis, *R*_ct_ is the electron transfer resistance at the electrode/electrolyte surfaces proportional to the diameter of the semicircle in the mid-range frequencies and indicates electrochemical kinetic-limited behavior, and CPE is the constant phase element that can be applied whenever there is a non-ideal electrochemical system. The following formula can be used to measure the impedance of a constant phase element: ZCPE = |*T*(*jω*)^*P*^|^−1^, where *T* represents capacitance characteristics and corresponds to the specific surface area and *P* is an exponent factor that can be ascribed to the roughness of the electrode, with values of between 0 and 1. *n* = 1 is used for an ideal capacitor, which is rarely the case for real systems, while *n* = 0 and 0.5 represent the physical resistance and Warburg elements, respectively. *ω* is angular frequency. *T* and *P* do not change upon changes in frequency. However, based on the fitted circuit used for the Nyquist curves, the CPE element reflects the double layer capacitance of the composite electrode and is used instead of *C*_dl_ to better show the inhomogeneity of the electrode surface. Based on the aforementioned explanations, the composite material shows lower *R*_s_ values rather than other electrodes, as revealed in the inset in Fig. 14. It can be seen that the GO/NF electrode shows the lowest charge transfer resistance followed by the Co(OH)_2_/NF and Co(OH)_2_/p-rGO/NF electrodes. This is due to the better electrical conductivity of this electrode in comparison to the other electrodes. Hence, the synergism effects among the components within the composite material result in a decrease in the charge transfer resistance, an increase in the interfacial active surface area and an improvement in the electrical conductivity of the material in comparison to the other two electrodes. The composite material shows lower final impedance along with a shorter line than the other electrodes, which can be ascribed to facilitated electrolyte ion diffusion through the composite material in comparison to the other prepared electrodes. The higher electrochemical performance of the Co(OH)_2_/p-rGO/NF electrode in comparison to the cobalt hydroxide/NF electrode originates from several factors, which are: (i) the presence of a carbonaceous material with a high specific surface area and electrical conductivity, (ii) the lower agglomeration of the composite electrode in comparison to the cobalt hydroxide electrode, (iii) facilitated electron and electrolyte ion transfer within the composite electrode and, finally, (iv) better structural stability due to the presence of carbonaceous material in the composite electrode. All of these factors make the composite electrode featuring Co(OH)_2_ and p-RGO an attractive potential candidate for use in supercapacitor applications. Furthermore, it is hoped that the simple fabrication strategy will encourage researchers to use this method for the preparation of any type of composite electrode, including a carbonaceous material and transition metal hydroxides/oxides, for use in energy storage applications. It is worth noting that in the composite material, p-rGO acts as both a binder and electrically conductive support to prevent the agglomeration of deposited hydroxide material and increase the conductivity of the composites, leading to an efficient redox reaction related to the active material at the electrode/electrolyte interface. Notably, it should be mentioned that a great improvement in the interfacial surface area affirms the facile electrolyte accessibility to the interior portions of the composite, which could meaningfully decrease the length of the ionic transport pathways that are necessary to deliver high capacitance.

#### 3.3.2. Two-electrode system

**Fig. 15:**
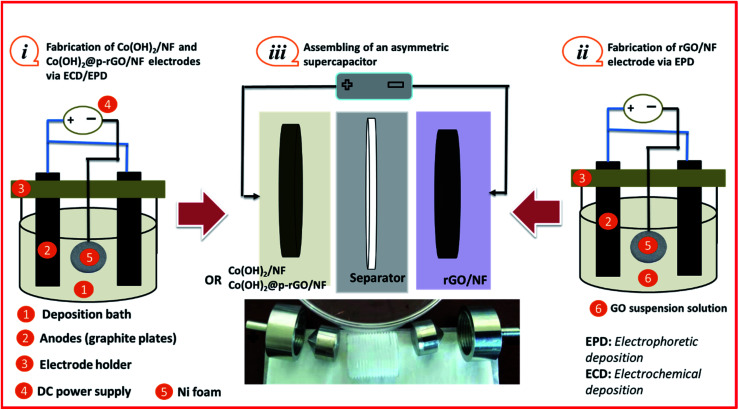
Schematic of the fabrication and assembly of an asymmetric supercapacitor (ASC): (i) fabrication of the positive electrode, (ii) rGO/NF fabrication as the negative electrode and (iii) structure of the assembled ASC device.

Fig. 15 shows a schematic view of the assembly of an asymmetric supercapacitor (ASC) device using the negative/positive electrodes. Notably, the rGO/Ni foam is the negative electrode and the Co(OH)_2_@p-rGO/NF or Co(OH)_2_/NF is the positive electrode. Before the construction of each ASC device, its corresponding single electrodes were fabricated *via* electrochemical deposition (ECD) or electrophoretic deposition (EPD) techniques.

First, Co(OH)_2_ was deposited onto coin-shaped NF (surface = 2 cm^2^) *via* a cathodic ECD technique (Fig. 15i). The Co(OH)_2_@p-rGO/NF electrode was also fabricated *via* an EPD-ECD process (Fig. 15i), as mentioned in Section 2.3.

**Fig. 16:**
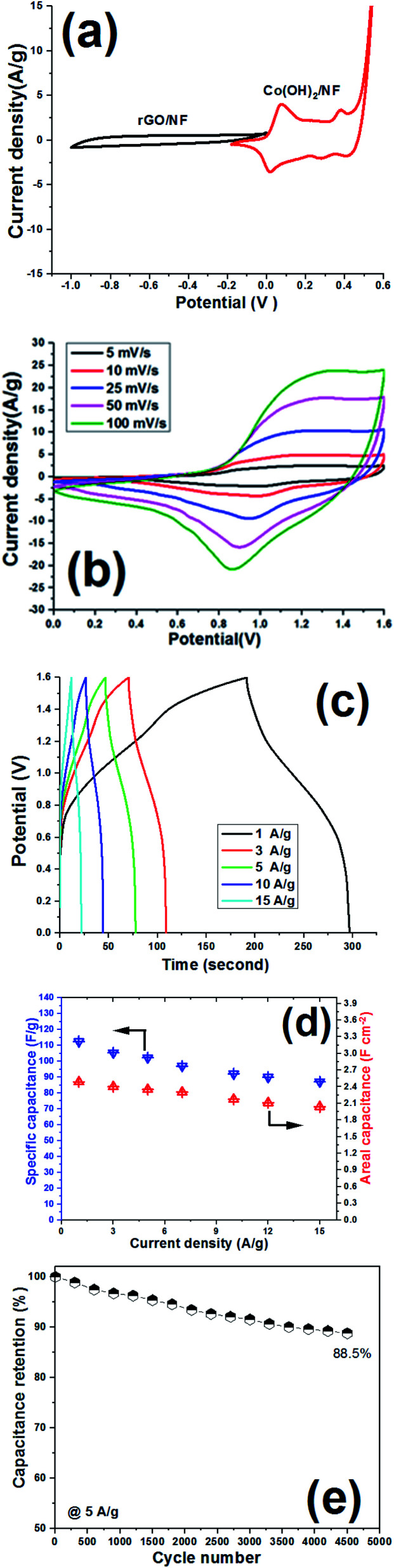
Electrochemical performance of the Co(OH)_2_/NF//rGO/NF ASC device. (a) CV curves of the Co(OH)_2_/NF and rGO/NF electrodes at a scan rate of 10 mV s^−1^, (b) CV curves of the assembled ASC device at different scan rates, (c) its GCD curves at different current densities, (d) the calculated specific capacitance *vs.* current load, and (e) the capacitance retention *vs.* cycle number at 5 A g^−1^.

The negative electrode was chosen to be rGO deposited onto Ni foam, *i.e.* rGO/NF. This electrode was fabricated by direct EPD of rGO onto NF from a GO dispersion electrolyte, as schematically shown in Fig. 15(ii). Then, two different types of electrodes were pressed together as a face-to-face structure with a PVA separator in the middle, and two ASCs were assembled, as schematically shown in Fig. 15(iii), named Co(OH)_2_/NF//rGO/NF and Co(OH)_2_@p-rGO/NF//rGO/NF ACS. Fig. 16a shows CVs of the fabricated electrodes at a scan rate of 10 mV s^−1^. The rGO/NF negative electrode exhibits well-defined EDL capacitance behavior over an applied potential window of −1 to 0 V (as seen in Fig. 16a). Furthermore, the fabricated Co(OH)_2_/NF electrode shows faradaic redox storage behavior at potentials of −0.2 to 0.6 V (Fig. 16a). The CV curves of the fabricated ASC device using rGO/NF and Co(OH)_2_/NF electrodes (*i.e.* Co(OH)_2_/NF//rGO/NF) were recorded over a potential range of 0–1.6 V, with the obtained results shown in Fig. 16b. This assembled ASC device exhibits pseudocapacitive behavior. The GCD curves of the ASC device recorded at different current densities of 1 A g^−1^ to 15 A g^−1^ are shown in Fig. 16c. Using the GCD data and eqn (3) and (4), the specific capacitances of the Co(OH)_2_/NF//rGO/NF device were calculated to be 122.5, 105.4, 102.3, 97, 92.1, 80 and 77.1 F g^−1^ at current loads of 1, 3, 5, 7, 10, 12 and 15 A g^−1^, respectively (Fig. 16d). Furthermore, areal capacitances of this ASC device were measured to be 2.486, 2.401, 2.35, 2.302, 2.174, 2.11 and 2.04 F cm^−2^ at current loads of 1, 3, 5, 7, 10, 12 and 15 A g^−1^, respectively (Fig. 16d).

The cycling ability of the fabricated Co(OH)_2_/NF//rGO/NF ASC device was also examined at a current density of 5 A g^−1^, and it was observed that this device preserved 88.5% of its initial capacitance after 4500 cycles (Fig. 16e). The energy density and power density of the device were also calculated using eqn (5) and (6). It was obtained that the Co(OH)_2_/NF//rGO/NF ASC device can deliver an energy density of 41.9 W h kg^−1^ at a power density of 684 W kg^−1^. This data thus shows the good capacitive ability of the Co(OH)_2_/NF//rGO/NF ASC device.

**Fig. 17:**
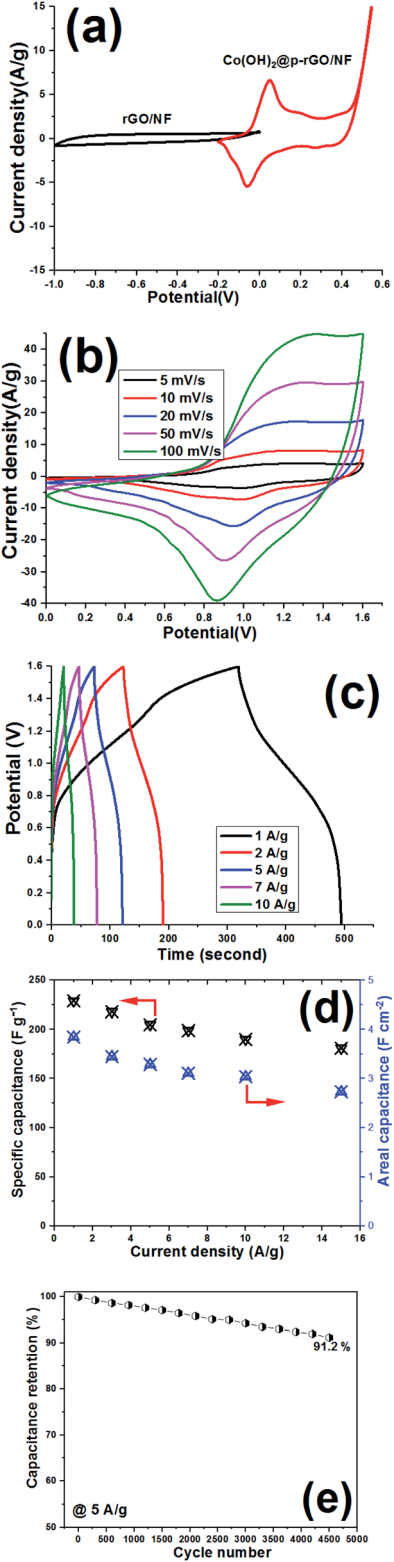
Electrochemical performance of the Co(OH)_2_@p-rGO/NF//rGO/NF ASC device. (a) CV curves of the positive and negative electrodes at 10 mV s^−1^, (b) CV curves of the ASC device at different scan rates, (c) its GCD curves at different current densities, (d) the calculated specific capacities *vs.* current density, and (e) capacitance retention as a function of cycle number over 2500 cycles in a two-electrode system.

The electrochemical performance of the Co(OH)_2_@p-rGO/NF//rGO/NF ASC device was also analyzed by conducting CV, GCD and long-term cycling tests, with the results of these tests given in Fig. 17. CV curves of the rGO/NF and Co(OH)_2_@p-rGO/NF electrodes recorded at a scan rate of 5 mV s^−1^ are shown in Fig. 17a. Suitable charge storage behavior is observed for both the negative and positive electrodes. In fact, a pair of faradaic redox reactions play main roles in the CV profile of the fabricated Co(OH)_2_@p-rGO/NF at potentials of −0.2 to 0.6 V (Fig. 17a).

The fabricated Co(OH)_2_@p-rGO/NF//rGO/NF ASC device was analyzed using CV techniques, and its CVs were recorded at potentials in the range of 0–1.6 V, as shown in Fig. 17b. At all applied scan rates in the range of 5–100 mV s^−1^, redox reaction peaks can be clearly seen for the ASC device. Also, GCD profiles of the Co(OH)_2_@p-rGO/NF//rGO/NF ASC device are shown at different current densities (Fig. 17c), with the specific capacitances calculated using eqn (3) and (4), with the results shown in Fig. 17d. It was found that the fabricated Co(OH)_2_@p-rGO/NF//r-GO/NF ASC device exhibits specific capacitances of 229, 218, 205, 199, 190 and 181 F g^−1^ under applied loads of 1, 3, 5, 7, 10 and 15 A g^−1^, respectively (Fig. 17d). These data reveal that this ASC device retains 79.1% of its initial capacitance at a high current density of 15 A g^−1^ (*i.e.* 181 F g^−1^), verifying its good rate performance. Furthermore, the fabricated Co(OH)_2_@p-rGO/NF//rGO/NF ASC device shows areal capacitances of 3.82, 3.44, 3.29, 3.11 and 2.83 F cm^−2^ at 1, 3, 5, 10 and 15 A g^−1^, respectively (Fig. 17d). For this assembled ASC device, a cycling retention of 91.2% is observed during 4500 cycling at 5 A g^−1^ (Fig. 17e). Using eqn (5) and (6), the energy density and power density of the fabricated Co(OH)_2_@p-rGO/NF//rGO/NF ASC device were calculated to be 68.7 W h kg^−1^ and 895 W kg^−1^, respectively. Overall, the results of the two-electrode tests with fabricated electrodes show the good performance of Co(OH)_2_@p-rGO/NF as a positive electrode for use in an ASC device.

## 4. Conclusions

A simple electrochemical procedure was developed for the one-step co-deposition of a cobalt hydroxide/porous rGO composite onto a 3D nickel foam support. The fabricated composite was found to have a crystalline morphology of β-Co(OH)_2_ nanoplates grown on p-rGO nanosheets. The high surface area, crystalline structure and porous plate texture of the deposited composite were confirmed from BET, XRD, FT-IR, FE-SEM and TEM data. Electrochemical studies, including CV and GCD measurements, confirmed the excellent supercapacitive performance of the fabricated Co(OH)_2_/p-rGO nanocomposite, where the composite electrode still delivers capacitance values of 1424 F g^–1^ and 1085 F g^–1^ after the 6000th cycling of the material under applied current densities of 5 A g^–1^ and 15 A g^–1^, respectively. Furthermore, the composite material retains more than 75% of its initial capacitance upon increasing the current density from 1 to 30 A g^–1^, while the cobalt hydroxide electrode only retains around 29% of its initial value, reflecting the effect of the addition of the porous rGO as a conductive support. The energy density and power density of the fabricated Co(OH)_2_@p-rGO/NF//rGO/NF ASC device were calculated to be 68.7 W h kg^−1^ and 895 W kg^−1^, respectively. The developed method could thus open up a new avenue for the one-step fabrication of metal hydroxide-carbonous nanomaterials in a short time that has the potential to be scaled up.

## Conflicts of interest

There are no conflicts to declare.

## Supplementary Material
